# Electrophysiological analysis of mesenchymal stem cells post-cryopreservation highlights the need for a recovery period: implications for cell-based therapies

**DOI:** 10.1186/s13287-025-04850-0

**Published:** 2025-12-20

**Authors:** Matthew P. Johnson, Muhammad Hamza Tariq, Michael Pycraft Hughes, Nupur Kohli

**Affiliations:** 1 Department of Biomedical Engineering and Biotechnology, Khalifa University of Science and Technology, Abu Dhabi, UAE; 2 Healthcare Engineering Innovation Group, Khalifa University of Science and Technology, Abu Dhabi, UAE

**Keywords:** Cryostorage, Electrophysiology, Zeta potential, Membrane potential, Dielectrophoresis

## Abstract

Human mesenchymal stem cells (MSCs) are characterized by their ability to differentiate into a variety of cell types, including osteocytes, chondrocytes, and adipocytes, making them promising candidates for cell-based therapies. Whilst the optimum method of clinical use is to use MSCs immediately after harvesting and expansion, there is often a need to cryostore MSCs before transplantation; this negatively impacts MSCs, affecting phenotypic marker expression, viability, differentiation potential, and other properties. There is consequently a requirement for methods to determine the biophysical state of MSCs post-thaw, in order to determine an optimum time for implantation after cells have recovered to a “normal” state. Typically, the primary method of assessing this is by measurement of cell viability; the cellular membrane is one of the key indicators of cell health and cell-cell interactions. Membrane-integrity dyes such as trypan blue are commonly used for binary viability checks, and ion tracking dyes offer insight into channel activation. However, these are typically expensive and time-consuming to use, limiting their efficacy in relatively high-throughput manufacturing scenarios. We used novel electrophysiological methods to assess MSC-health following freezing and thawing. Our results indicate that MSC health deviates significantly from its original phenotype immediately after thawing and only begins to resemble the pre-freezing state after three days. Notably, cell membrane capacitance does not fully recover to pre-freezing levels, even after this period. Results also suggest that the use of DMSO as a cryopreservant may be associated with the prolonged recovery period.

## Introduction

Human mesenchymal stem cells (MSCs) are multipotent cells derived from mesodermal lineages, characterized by their ability to differentiate into a variety of cell types, including osteocytes, chondrocytes, and adipocytes [1]. The ability to differentiate into cells whose loss is associated with a range of diseases (particularly those associated with old age) make MSCs promising candidates for a wide range of cell-based therapies (CBTs) [2] where living cells are (re)implanted into patients for therapeutic benefit. The existence of cells capable of acting as precursors to osteocytes and other bone-associated cells was first described by Freidenstein in the 1960s; however, the cells were not subject to detailed study until the 1990s, beginning with Caplan’s landmark study in 1991 which introduced the name [4, 7, 8]. Since then, the potential therapeutic benefits MSCs has been the subject of extensive scrutiny, with several interventions reaching clinical trials for a range of conditions.

However, to date only one MSC-based therapy has been approved by the US Food and Drug Administration (FDA) for the treatment of pediatric steroid-refractory acute graft-versus-host disease (SR-aGVHD). This can be attributed to several challenges, including a lack of standardized protocols for isolation, characterization, and manufacturing of MSCs, as well as inconsistent clinical outcomes across trials. These issues have hindered the development of reliable, scalable MSC-based treatments, contributing to delayed FDA approvals; as of 2025, only 37 CBTs have been approved for conditions including multiple sclerosis, sickle cell anaemia, diabetes, Crohn’s disease and Graft vs. Host disease [9].

To achieve FDA approval, a new therapy must pass a number of regulatory stages. First, early screening is conducted in animal models [10]. Of successful preclinical animal trials, only about 37% transition to Phase 1 clinical human trials [11]; between 2019 and 2023, this equated to around 230 phase 1 trials per year [12]. After entering clinical trials only around 17% of CBTs reach Phase 3 (after proving both safety and efficacy), and less than 10% achieve approval [13]. The failure of 83% of CBTs to pass Phase 2 demonstrates either a biological incompatibility between the preclinical model and human patients, or a methodological failure resulting from alterations to method when translating from preclinical to clinical applications [14–16].

A key cause of many of the failures of CBTs to translate from preclinical study to clinical trials is the requirement for cells to be stored before therapeutic use. Ideally, cells should be transplanted immediately after harvesting and expansion; however, for clinical therapeutic use, it is necessary to expand cells and then store them until the patient is ready to receive them, and the clinical team is available to perform the transplant. Consequently, cryostorage is used to store cells until required.

However, cryostorage has been shown to impact MSCs in multiple ways, including phenotypic marker expression, viability, differentiation potential, and other functional properties. While some effects are transient and recover over time, others may introduce variability that necessitates optimized cryopreservation protocols to ensure the efficacy of MSC-based therapies [10–21]. Although most studies do not report changes in differentiation or cell growth when assessed in vitro, there are conflicting findings that question whether MSC phenotypes and bioactivity are affected by the cryostorage process. Conversely, when assessed in vivo, studies suggest it causes changes in bioactivity, whilst distribution in the patient is altered, engraftment efficiency is reduced, and patients report an increase in adverse effects [13].

It is therefore important to assess the condition of MSCs post-thaw to ensure that crystored cells have returned to a sufficiently “normal” pre-frozen state before implantation [19–21]. The standard method of assessing this is by measurement of cell viability. Cellular membrane integrity is a key indicator of cell viability, and membrane-integrity dyes such as trypan blue are commonly used for binary viability checks. However, whilst these methods can indicate cell death, they do not report altered function in the living cell. Conversely, ion tracking dyes offer insight into ion channel activation to investigate ionic transfers across membranes, but these are typically expensive and time-consuming to use, limiting their efficacy in relatively high-throughput manufacturing scenarios. Consequently, there is a need for alternative methods to assess not only viability, but physiological state. Such methods should be both simple to perform, and low-cost, in order to fit standard clinical workflow.

Whilst many markers of cell physiology are available, most require expensive tools such as flow cytometers, and expensive biological indicators such as fluorescent markers. In order to find an alternative method, we considered electrophysiology as an alternative approach to measuring cell physiological state. Whilst conventional electrophysiology relies on slow, complex and expensive methods such as patch clamp or microelectrodes [22, 23], or reporters such as fluorescence or radioactive tracers which allow relative, rather than direct, measurement [24, 25], other methods offer rapid analysis time and low operating costs. For example, two novel methods of electrophysiological measurement that allow rapid, direct measurement of cell electrophysiology at low cost are Dielectrophoresis (DEP) [26] and zeta potential measurement using electrophoretic light scattering (ELS) [27]. Both methods measure cell movement in response to external electrical fields, but use types different electric fields (uniform vs. non-uniform, alternating-current vs. direct current). DEP is mechanistically related to the more common biomedical tool of electrophoresis, but uses the measurement of induced motion of cells suspended in a non-uniform alternating electric field to determine a range of electrical properties. These are obtained by fitting the measured behaviour of cell ensembles as a function of applied field frequency. This yields a “DEP spectrum” that can be analysed to extract the mean values of cytoplasm conductivity *σ*_*cyto*_, as well as whole-cell membrane conductance *G*_*wc*_ and capacitance *C*_*wc*_ [26, 28]. Moreover, analysis of *σ*_*cyto*_ at multiple extracellular ion concentrations with corresponding conductivity *σ*_*med*_ has been shown to be an effective method of estimating mean cell resting membrane potential *V*_*m*_ [29]. DEP has been used in a variety of cell biological applications; for example, it has been used to identify differentiation fate in human neural stem cells [30, 31], discriminate between mesenchymal stem cell lineages [32–35], and track the changes in electrophysiology of chondrocyte corresponding transition to fibroblasts in culture [36]. DEP is sufficiently rapid to allow time-sensitive measurements, and has been used to assess circadian rhythms in adipocytes [37] and red blood cells [38]. Others have used DEP to study the effects of storage on red blood cells [39, 40] and cells found in urine samples [41] and tuberculosis infection in macrophages [42] as well as application as a clinical diagnostic for oral cancer [43, 44] and bladder cancer [45].

**Fig. 1:**
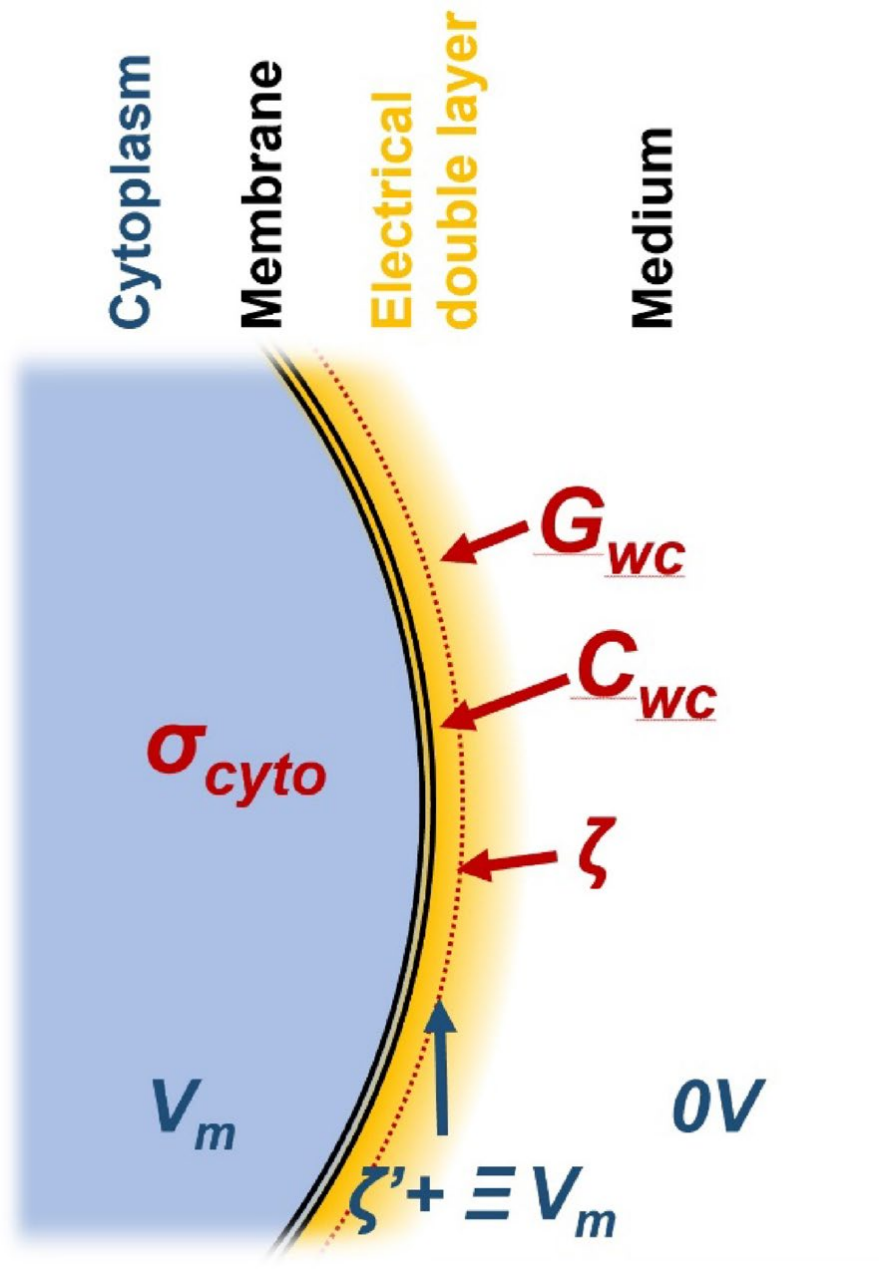
Outline of the locations for charges about the cellular membrane, including *V*_*m*_ and ζ, and indications of the radial locations of the DEP-derived whole cell capacitance and conductance

The zeta potential (or ζ-potential) is a well-characterised property of particles, both organic and inorganic, in contact with an aqueous medium. Any object in such a medium (including cells and proteins, but also electrodes and cuvettes) that carries a charge on its surface will attract countercharge (typically cations) from the medium. This layer of countercharge partially screens the surface potential; at the surface, ions become hydrodynamically trapped, such that the potential at the outer perimeter of this “stagnant layer” (the ζ-potential) effectively defines how the surface interacts electrostatically with its environment. The ζ-potential plays an important role in adhesion, coagulation and flocculation; it is the reduction in ζ-potential that causes milk to curdle on the addition of lemon juice [27]. It has also been shown [46] that ζ-potential is affected by membrane potential such that a portion of *V*_*m*_ is added to ζ, such that it does not arise simply from the fixed surface charge but gives a reflection of the internal cellular state. This was exploited by Chacar et al. [47] to measure depolarisation of cardiomyoblasts, for example, and has been implicated in biological process from developmental biology to cancer [48]. The parameters determined by DEP and ELS are shown schematically in Fig. 1.

In this paper we use DEP and ELS to observe changes in cell electrophysiology of MSCs following freezing and thawing. Results suggest that the electrophysiology of MSCs changes immediately after thawing, but these properties more closely align with the original phenotype 74 h post-thaw (although *C*_*wc*_ does not fully return to its original level). This suggests that DEP and ELS may provide a useful, rapid method for assessing cell viability, and also implies that a recovery period of approximately three days post-thaw may be necessary for cells to return to a near-fresh state before implantation.

## Results

### Cell radius, viability, and number

The model for extraction of electrical parameters from DEP spectra requires the mean cell diameter to be measured. Cell radii were measured before and after cryopreservation; pre- and post-cryo preservation, the viability remained between 96 and 98% throughout the experiment; similarly, cell diameter remained largely constant throughout all measurements, being 15.1 ± 0.9 μm for viable cells and 13.6 ± 0.6 μm for dead cells. Examination of the DEP spectra of post-thaw samples showed no evidence of multiple populations and were modelled as single populations using the live cell radius.

When overall cell numbers were analysed, we found that cell counts declined after thawing, reducing further at 26 h before increasing at 50 h and increasing further at 74 h. Numbers did not recover to the pre-freeze level by the end of the experiment, with cell counts at the end of the experiment being 54% of the pre-freeze value. This suggests that simple binary live/dead assays may misrepresent a condition where cells affected by the freezing process are not merely damaged, but destroyed.

### Passive electrical properties

**Fig. 2:**
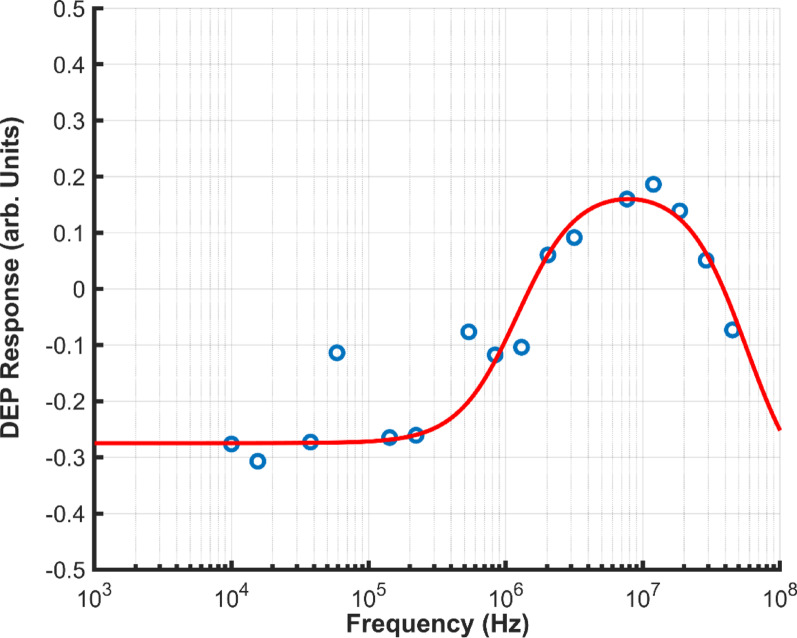
Example of fitting the DEP spectra (red line) to 3DEP data (blue circles). Sample shown is ‘Prefreeze’ donor B in 144 mS/m media

**Fig. 3:**
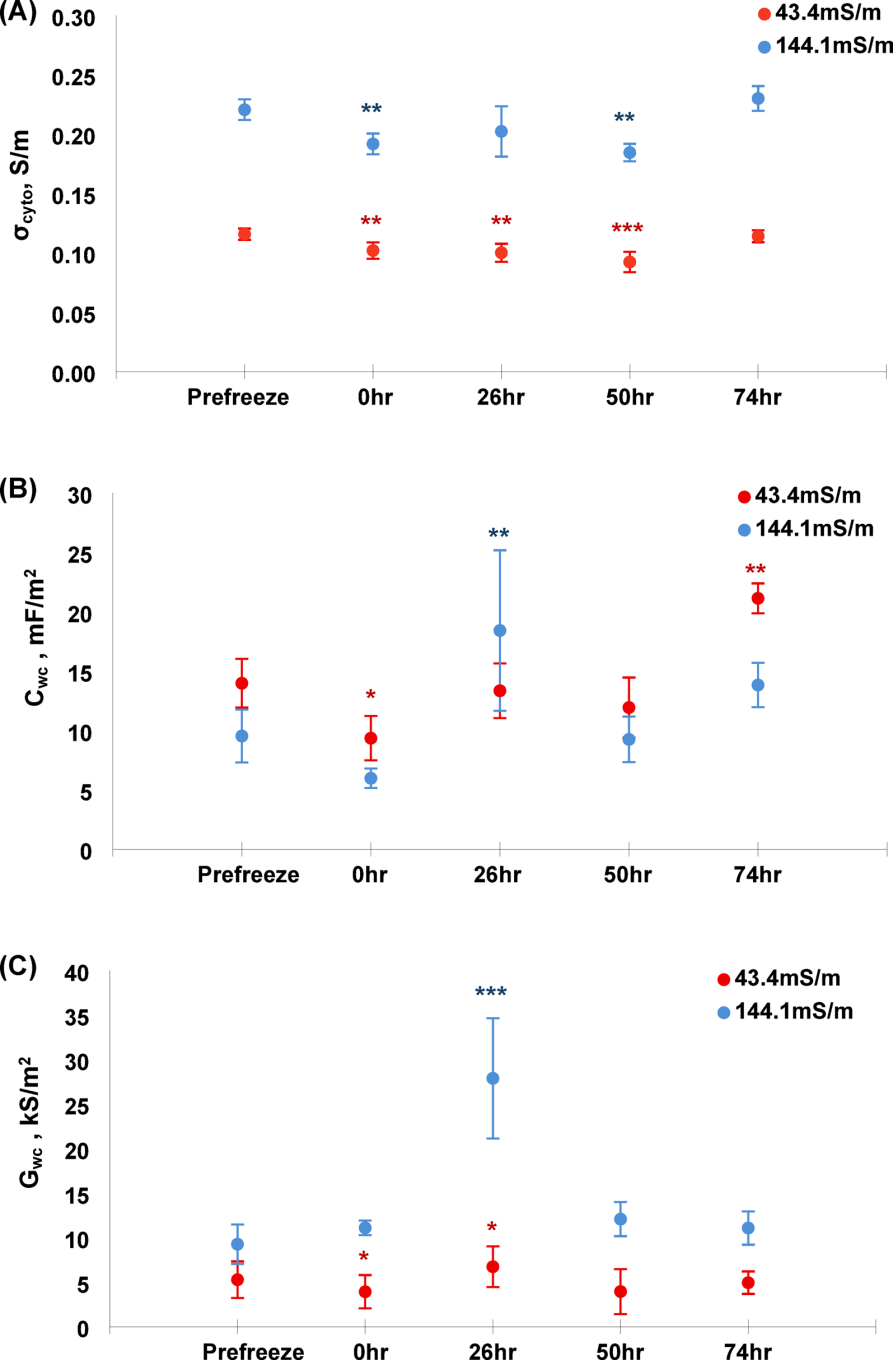
(**A**) Cytoplasmic conductivity, (**B**) Effective whole cell capacitance, and (**C**) whole cell conductance results. All plots display both medium conductivities separately, error bars denote standard errors and significance from ‘Prefreeze’ within conductivities are denoted by asterisks

DEP analysis yielded spectra such as the example shown in Fig. 2 (points). This was analysed using a best fit model (line) derived from estimated best-fit electrical parameters. These were extracted for each experiment. The results across all experiments can be seen in Fig. 3, which features both the results and the illustration of statistically significant differences.

Considering membrane capacitance *C*_*wc*_ first, we observed a wide range of values at each timepoint, leading to large standard deviations. Statistical significance was observed between baseline and days 1 (where the value was lower than baseline) and 3 (where it was higher than baseline); it was also observed to be higher at day 1 at the higher conductivity. However, the degree of variation suggests that it is unlikely to be used as a biomarker.

The cytoplasm conductivity *σ*_*cyto*_ showed statistically significant differences against baseline within each conductivity group, with statistically significant reductions values of conductivity at day 0, day 1 and (for 43 mSm^− 1^ only) day 2 before returning to baseline at day 3. Finally, in membrane conductance *G*_*wc*_, values remained broadly in line with baseline throughout the experiment with two notable deviations; both conductivities showed statistically significant differences to their respective baselines at day 1, where *G*_*eff*_ was elevated in both cases. Statistical significance was also observed at the lower conductivity at time 0 h.

### Membrane and zeta potential

**Fig. 4:**
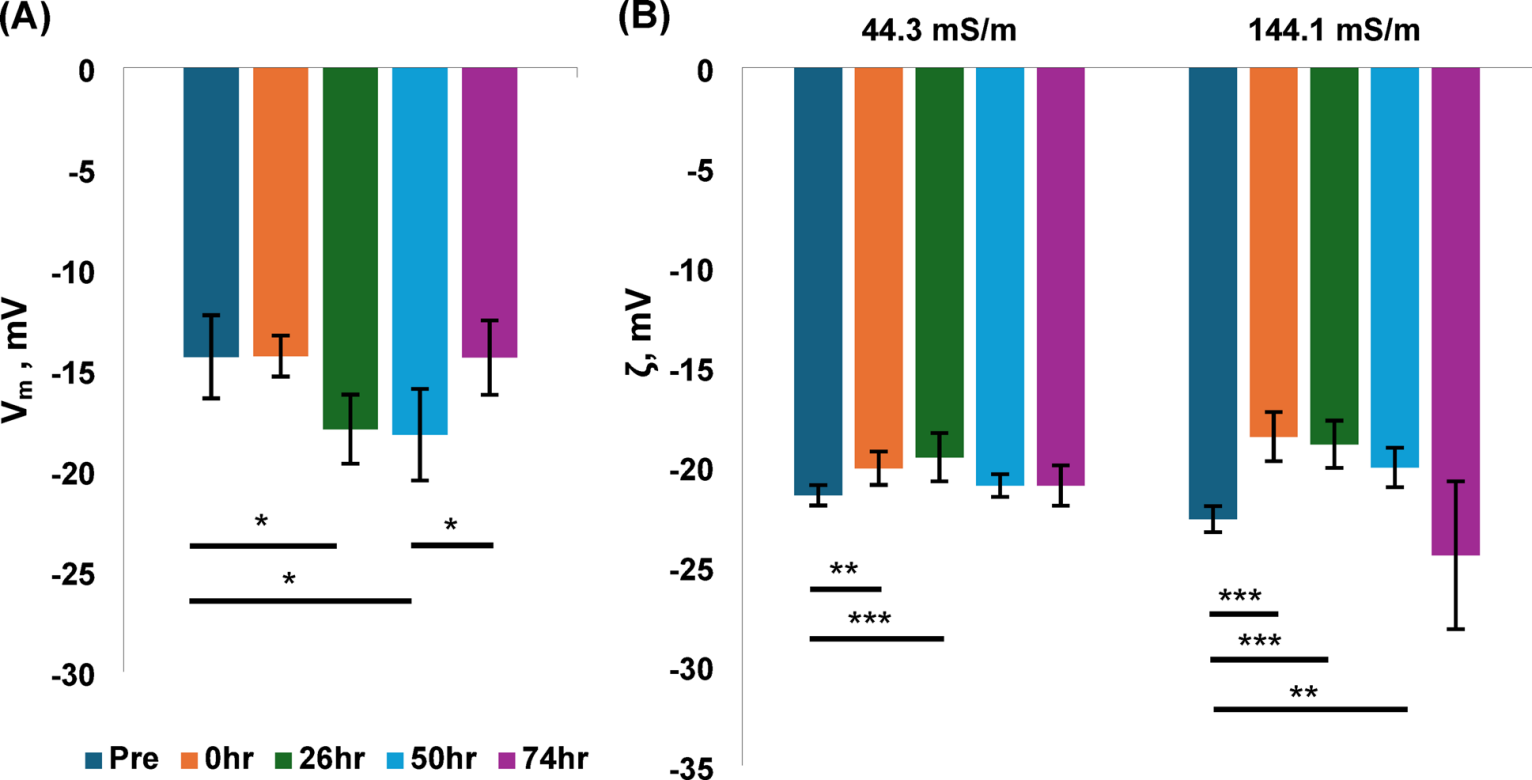
**A** Membrane potential results. **B** ζ-potential results by medium conductivity. Error bars denote standard errors, significance shown only within like conductivities, only significant relationships labelled

The ζ-potential and derived values of *V*_*m*_ are shown in Fig. 4. We used the change in *σ*_*cyto*_ to estimate *V*_*m*_. Work by Hughes et al. [28] showed that *V*_*m*_ can be calculated using the change in *σ*_*cyto*_ for different values of *σ*_*med*_:

1 $$\:{V}_{m}\:=\:\frac{RT}{F}\mathrm{l}\mathrm{n}\left(\frac{\delta\:{\sigma\:}_{med}}{{\delta\:\sigma\:}_{cyto}}\right)+{\varPsi\:}_{ext}$$

Where *R* is the gas constant, *T* is temperature in Kelvin, *F* is the Faraday constant, *δσ*_*cyto*_ is the measured change in cytoplasm conductivity for a change in medium conductivity *δσ*_*med*_, and *Ψ*_*ext*_ is the cell surface potential, measured to be -12 mV across a wide range of cell types. Applying this to our *σ*_*cyto*_ data at 43 mSm^− 1^ and 143 mSm^− 1^ yielded the membrane potential data shown in Fig. 4a. When we used the *σ*_*cyto*_ data to calculate *V*_*m*_, we found statistical differences compared to baseline after 26 h and 50 h; in both cases *V*_*m*_ was hyperpolarised compared to *V*_*m*_ before freezing. No differences were observed either immediately after freezing, or at 74 h.

We also measured the ζ-potential (Fig. 4b). This is affected by many factors, including medium conductivity; elevation of charge in the medium reduces the size of the electrical double layer, and consequently lowers the ζ-potential (which is measured at a fixed distance from the cell surface, and hence is closer to the bulk medium at higher conductivity). It is also, as described previously, related to *V*_*m*_. When we analysed the ζ-potential of cells in 43 mSm^− 1^ solution, we found strongly significant differences between pre-stored cells and those measured both immediately after thawing (*p* < 0.01) and 26 h (*p* < 0.001) at low conductivity. When the cells were analysed at higher conductivity, significance was observed immediately after thawing (*p* < 0.001), 26 h later (*p* < 0.001), and 50 h after thawing (*p* < 0.01). No differences were observed after this at either conductivity, suggesting the normal (pre-freeze) values has been established. All the observed differences corresponded to depolarisations compared to baseline.

## Discussion

The nature of stem cells, as progenitors for a wide range of cell types, offers great therapeutic benefits for many diseases. However, since these cells must be relied upon to act in ways that are both anticipated and therapeutically beneficial without further post-implantation interventions, it is important that the transplantation process be performed with cells that are as close as possible to their normal physiological state. Whilst many measurements of stem cell physiology post-cryopreservation have been reported, these often involve expensive, time-consuming processes such as flow cytometry to determine that state; whilst these processes can be effective and enlightening, they are often too cumbersome to be used routinely as part of clinical workflow in a busy hospital. This is particularly the case where cells may need to be monitored multiple times before being considered suitable for transplantation.

The electrophysiological methods explored here offer new mechanisms for rapidly measuring cell state without the need for expensive fluorescent labels or time-consuming cell preparation. Both methods rely on a simple resuspension followed by analysing using a disposable cuvette, and neither method requires significant training to learn or interpret data, meaning that either would be easily inserted into a clinical workflow. Whilst the measurement of radius step was included here in order to extract absolute electrical parameters from the DEP spectra, other analysis methods exist that provide simple binary discrimination without including this step, thereby speeding up clinical workflow. For example, several studies have employed the Mean Difference Value (MDV) as a method of reducing the spectrum to a single index number that can be used for diagnostic purposes [43, 44].

### Comparison to other studies

Our results suggest that both ζ and *V*_*m*_ may be indicative of cell health and MSC recovery after cryopreservation. Whilst significant changes were observed in the earlier stages of recovery, it was notable that all parameters except membrane capacitance had returned to baseline 76 h after thawing, with no statistically significant differences observed in ζ-potential, *V*_*m*_, *σ*_*cyto*_ or *G*_*eff*_.

Most studies of the effects of cryopreservation on MSCs have focussed on medium-to-long term measurements, primarily focussing on cell counts and differentiation potential. For example, Vasconcelos et al. [21] reported no change in cell number after thawing; however, once cells began dividing, there was an increase in cell numbers from 24 h onwards similar to that observed by us. Similarly, Dulugiac et al., [20] reported no changes in histology and immunohistochemistry in cells before and after cryopreservation, particularly with regards to differentiation potential, whilst Bruder et al. also reported no long-term effects on cell doubling rate or differentiation potential [23] compared to fresh cells from the same donor. Zhang et al. [49] reported similar growth rates and no change in surface markers or cell morphology, but an altered differentiation profile.

However, consistent with our findings, shorter-term studies of cells immediately after removal from cryopreservation have noted differences in cell behaviour, amounting to what was described by Moll et al. [50] as a “cryo stun” effect. This is particularly notable in the first 24 h after transplantation, where Irioda et al. [51] described decreased DC49d expression, colony formation and viability, and Bahsoun et al. [52] described reduction in metabolic activity, cell adhesion potential and metabolic activity, whilst Antebi et al. [53] reported a similar 24 h period during which cells showed reduced immunomodulatory function and surface marker expression. We also observed a decline in cell viability immediately post-thawing, which could be attributed to cryo-induced apoptosis, osmotic stress, and membrane damage during the freeze-thaw cycle, which has also been previously reported by Mamo et al., and Tan et al. [54, 55]. Moreover, in the study by Bahsoun et al. [52], the researchers suggested that cell numbers did not recover until the third day after thawing. As reviewed by Galipeau and Sensébé [10], thawed MSCs exhibit “signatures of cell injury” in the first 24 h post-thawing, including loss of cell function and increased likelihood of attack by immune cells, indicating a need to identify when cells have regained a normal phenotype ahead of transplantation, a point reinforced by Cottle et al. [17] in a review of cryopreservation methods. All of these point to the need to evaluate the readiness of MSCs for successful transplantation; the estimation of normalcy returning at 76 h is in line with these studies.

### Potential origin of electrophysiological effects

We may also speculate on the reason for the observed differences, and whether these differences arise from the cells themselves, or from the cryopreservation process.

There are few changes from baseline in *G*_*wc*_, suggesting that this is largely unaffected by the cryopreservation process. Conversely, *C*_*wc*_ changes throughout the process up to and including the final timepoint, suggesting that there are long-term phenotypic alterations in membrane folding which do not impact on cellular function, but which nevertheless indicate potentially far-reaching effects of cryopreservation on cell behaviour.

One additional point of interest is the interplay of the two measured electrical potentials. It has been shown in prior studies [51] that DMSO has an impact on the electrical properties of cells—specifically, on the relationship between *Vm* and ζ-potential. This relationship was described by Hughes et al. [56] as following the Eq.

2 $$\:\zeta\:={\zeta\:}^{{\prime\:}}+\varXi\:{V}_{m}$$

Where ζ is the measured value of ζ-potential, ζ’ is the ζ-potential arising solely from fixed surface charge, and Ξ is a constant of proportionality describing the amount of *V*_*m*_ observed at the slip plane. Using red blood cells as a model, it was shown that DMSO reduces the value of Ξ from ca. 0.37 under normal conditions to below 0.1 [56], by preferentially binding to the cell surface and reducing the double layer capacitance [57].

As described in the Methods, the cryopreservation process includes the use of DMSO. This is an agent in widespread use for many purposes in cell biology, including as a cryopreservant (as here) and also as a carrier solution for molecules that are insoluble in water. It is known to have negative effects on cells, including reducing membrane thickness whilst increasing membrane permeability and fluidity [58]. It has also been shown to displace water molecules from the electrical double layer, even in small concentrations [59]. Since DMSO has a lower electrical permittivity than water [60], this has an impact on the electrical dynamics of the cells.

As DMSO was not used for the pre-frozen cells, and the expansion of cell numbers suggests that any DMSO carried over from the preservation process would either have been lost or distributed at low concentration across the cell population, we can assume that it plays no role in these cells but may affect the electrical properties immediately after thawing. This would explain why the ζ-potential is depolarised immediately after thawing, and at 26 h post-thaw compared to cells before freezing, since these would represent the cells affected by DMSO and consequently where the measured value of ζ broadly equates to ζ’ as described above.

If we use the post-thaw measured value of ζ-potential as indicating the baseline ζ’ and assume that this does not change (since it is primarily a function of charge on the lipid bilayer) and subtract this from the measured value of ζ, we obtain the magnitude of the component of ζ-potential arising from membrane potential (*ΞV*_*m*_); since we also have the DEP-measured value of *V*_*m*_, we can estimate the value of Ξ. Using ζ values measured at 143 mSm^− 1^, we obtain values for Ξ of 0.22 before freezing and 0.34 at 74 h, which are very similar to values published for cardiomyocytes [47] and red blood cells [56]. However, the estimated value of Ξ from thaw to 50 h inclusive was effectively zero (− 0.03 ± 0.05). This suggests that DMSO remains active in affecting cell electrical properties until the 74 h mark, and that the value of Ξ is broadly in line with that observed for other cells, particularly at the final timepoint.

The alteration of ζ-potential by DMSO may also be a factor in the efficacy of MSC-based therapies administered in the hours immediately following thawing, when cells retain the DMSO layer following cryopreservation. Since alteration of ζ-potential is known to alter cellular interactions (cell-cell, cell-ion and cell-protein) at the cell surface [61–63], this may interfere with the integration of cells in the patient, potentially disrupting the cell’s integration into its new environment as well as altering the body’s immune response to the injected cells. This potentially points to the necessity of developing cytopreservatives that do not interfere with cellular electrophysiology in order to better improve outcomes after administration.

## Limitations

While the study incorporates over five technical replicates, its findings are derived from tissue from only two donors; future work with additional donor samples will be important to validate and generalize these results. Furthermore, metabolic assays such as Alamar blue and MTT (3-(4,5-dimethylthiazol-2-yl)-2,5-diphenyltetrazolium bromide) assay, which are indirect measures of cell viability and health, may be incorporated in future experiments to comprehensively validate the findings of the electrophysiological parameters.

## Conclusion

We have presented two methods of electrophysiological analysis with potential for use in assessing the readiness of MSCs for transplantation. The DEP-based method indicated cell hyperpolarisation immediately after thawing that returned to baseline after three days; ζ-potential measurement showed similar effects but with no difference observed immediately after thawing, which we attribute to the presence of DMSO. Cytoplasm conductivity also showed a return to baseline by day 3, whilst membrane conductance showed a return to baseline by day 2. Only cell capacitance remained significantly different to the end of the experiment, suggesting long-lasting phenotypic changes remain present beyond the third day.

## Methods

### Cell culture

Following criteria of the International Society of cellular therapy (ISCT), cells were isolated and characterised, and sub-cultured in Dulbecco’s Modified Eagle Media-F12 (DMEM; Stem Cell Technologies, Canada), supplemented with 10% Fetal Bovine Serum (FBS; Gibco, USA) and 1% Gibco’s Penicillin–Streptomycin antibiotic solution. For analysis, UCMSCs (passage 7) were grown in T75 flasks with a seeding density of 10,000 cells/cm^2^. Cells were grown in incubators at 37 °C with 5% CO_2_ until 70–80% confluency, followed by preparing the samples for further analysis.

### Sample preparation

For cryopreservation and analyses post-thawing, 1 million cells per 1 ml of freezing media containing 10% DMSO (dimethyl sulfoxide) and 90% FBS were placed in cryovials in the IPA freezing system and kept at − 80 °C for 2 hours. This system decreases the temperature of cryovials by 1 °C/min. Following this, cryovials were moved to vapour-phase Liquid Nitrogen (LN) and stored for 48 h. Two days post storage in LN, cryovials were taken out and thawed at room temperature, followed by either conducting DEP analysis as 0 h post-freeze analysis or cultured in fresh media at the same seeding density of 10,000 cells/cm^2^ for three different time points analyses of DEP; 26 h, 50 h, and 74 h.

For DEP analysis, culture medium was aspirated, the cells were washed with Phosphate Buffered Saline (PBS; Gibco, USA) and incubated for 5–7 min at 37 °C and 5% CO2 with 1X trypLE solution (Thermo Fisher Scientific; USA) to facilitate cell detachment. After detachment, trypLE solution was neutralised with DMEM media. Cells were then pelleted by centrifugation at 500 g for 5 min at room temperature. The resulting pellet was washed by centrifugation in DEP media, an isosmotic sugar solution (8.5% w/v Sucrose, 0.5% w/v Dextrose, 250 µM MgCl_2_, 100 µM CaCl_2_) with conductivity adjusted to 43 mS/m by addition of Dulbecco’s Phosphate Buffered Saline (DPBS). After final suspension a cell count was performed to ensure a minimum viability of 90% with a total cell count of approximately 1 × 10^6^/mL, measured by automated trypan blue test (Invitrogen Countess III, Thermofisher, Massachusetts USA). The trypan blue tests automatically measured live and dead mean cell diameters, on average 452 cells per sample were used in the development of the DEP model, described below.

Samples were equally split, with one sample receiving another wash in the 43 mS/m DEP media, and the other being centrifuged and resuspended into DEP media with its conductivity raised to 144 mS/m by further DPBS.

*DEP measurement.* Cell electrophysiology measurements were taken using a DEPtech 3DEP (Heathfield, UK) [46]. 85 µL of cell solution was pipetted into 3DEP chips. These were analyzed at 20 frequencies, equally distributed logarithmically from 10 kHz to 45 MHz, for 30 s per technical repeat. After each analysis, the cells were purged and replaced with fresh cells. 5–6 technical repeats were conducted per sample. Suspended cells were treated as approximately spherical in shape, in line with standard DEP practice [26, 46–66]. The time-averaged dielectrophoretic force **F**_**DEP**_ of a spherical particle in a non-uniform electric field **E** is given by the equation [26]:

3 $$\:\mathbf{F}=2\pi\:{\epsilon\:}_{med}{{r}_{cell}}^{3}\mathrm{R}\mathrm{e}\left[K\left(\omega\:\right)\right]\nabla\:{E}^{2}$$

where *ε*_*med*_ is the absolute permittivity of the suspending medium, *r*_*cell*_ is the radius of the cell, ∇ is the gradient operator, *E* is the magnitude of the electric field, Re denotes the real part, and *K*(*ω*) is the relative polarizability of the particle given by the Clausius-Mossotti factor:

4 $$\:K\left(\omega\:\right)=\frac{{\epsilon\:}_{cell}^{*}-{\epsilon\:}_{med}^{*}}{{\epsilon\:}_{cell}^{*}+2{\epsilon\:}_{med}^{*}}$$

where the subscript *cell* refers to the whole cell, and *med* to the suspending medium. ε* is the complex permittivity, given by:

5 $$\:{\epsilon\:}^{*}=\epsilon\:-j\frac{\sigma\:}{\omega\:}$$

where *ε* is permittivity, *σ* conductivity, *ω* the angular frequency of the field, and *j* = √-1. In order to extend this to cells (which from a dielectric perspective are dominated by an insulating membrane and conducting cytoplasm) we can substitute a frequency-variant value of *ε*_*cell*_ in Eq. 4 that reflect this more complex structure. This “single shell model” combines the electrical properties of these using the following substitution [66]:

6 $$\:{\epsilon\:}_{cell}^{*}={\epsilon\:}_{m}^{*}\frac{{\left(\frac{{r}_{cell}}{{r}_{cell}\:-t}\right)}^{3}+2\frac{{\epsilon\:}_{cyto}^{*}-{\epsilon\:}_{mem}^{*}}{{\epsilon\:}_{cyto}^{*}+2{\epsilon\:}_{mem}^{*}}}{{\left(\frac{{r}_{cell}}{{r}_{cell}-t}\right)}^{3}-\frac{{\epsilon\:}_{cyto}^{*}-{\epsilon\:}_{mem}^{*}}{{\epsilon\:}_{cyto}^{*}+2{\epsilon\:}_{mem}^{*}}}$$

where *t* is the thickness of the membrane, and _*mem*_ and _*cyto*_ refer to the membrane and cytoplasm. This equation was fitted to the experimental data acquired from the 3DEP using a semi-automatic MATLAB fitting script developed by Tsai et al. [67] with limits and starting estimates shown in Table 1. The script calculated R^2^ values for each fit; a threshold value of R^2^ ≥ 0.6 was considered sufficient for inclusion of a technical repeat. Means and standard deviations were calculated for each DEP-derived electrophysiological property and sample. Technical repeats were combined and modelled separately for each biological repeat. Across samples for each timepoint, the resting membrane potential was determined from Eq. 1, using the measured change in cytoplasm conductivity δ*σ*_*cyto*_ from Eq. 4 for a measured change in medium conductivity δ*σ*_*med*_ [28, 65].

**Table 1:** Limits and starting estimates used for the fitting of single-shelled CM dielectrophoretic results

(units)	$\:{\epsilon\:}_{cyto}/{\epsilon\:}_{0}$	$\:{\epsilon\:}_{mem.}/{\epsilon\:}_{0}$	$\:{\sigma\:}_{cyto}$	$\:{\sigma\:}_{mem.}$
			(S/m)	(S/m)
Min. Limit	10	2	0.01	10^− 14^
Starting Estimate	50	15	$\:{\sigma\:}_{medium}$	10^− 5^
Max. Limit	160	200	1.6	10^− 2^

*Zeta potential measurement.* Zeta potential measurements were obtained with the Zetasizer Lab DLS system (Malvern Panalytical, UK). For each analysis 200 µL of the sample was diluted with 600 µL of the DEP media and injected into DTS1070 cuvettes. Zeta measurements were obtained 3–6 times (technical repeats) for each sample at each timepoint.

*Statistics.* For each timepoint, 3 samples were cultured in independent flasks for each of the 2 donors (*n* = 2). Between 3 and 6 technical-repeat measurements of zeta potential were captured for each sample, at each timepoint. Measurements were averaged across the six for each timepoint in each of the two media. Reported statistical significances (*p* < 0.05) were calculated from the pre-freeze values using two-tailed Z score tests.

## Data Availability

All data reported in this paper will be shared by the corresponding author upon request.

## Declarations

### Declaration of the use of AI

The authors declare that they have not use AI-generated work in this manuscript.
